# AXIN*2* rs2240308 polymorphism contributes to increased cancer risk: evidence based on a meta-analysis

**DOI:** 10.1186/s12935-015-0219-8

**Published:** 2015-07-04

**Authors:** Zhitong Wu, Yifan Sun, Shifu Tang, Chunming Liu, Shengbo Zhu, Lili Wei, Hong Xu

**Affiliations:** Department of Clinical Laboratory, Guigang City People’s Hospital, 1 Zhongshan Middle Road, Guigang, 537100, Guangxi People’s Republic of China; Department of Clinical Laboratory, Liuzhou Hospital of Traditional Chinese Medicine, 32 Jiefang North Road, Liuzhou, 545001, Guangxi People’s Republic of China; Department of Science and Education, Liuzhou Hospital of Traditional Chinese Medicine, 32 Jiefang North Road, Liuzhou, 545001, Guangxi People’s Republic of China

**Keywords:** AXIN2, Polymorphism, Cancer, Risk, Meta-analysis

## Abstract

**Background:**

Variants in the *axis inhibition 2 (AXIN2)* gene might alter the protein’s structure or function or create a multiprotein destruction complex in the Wnt signaling pathway and thus affect an individual’s susceptibility to cancer. The objective of this study is to evaluate broadly the evidence available for the *AXIN2* rs2240308 polymorphism and risk of cancer.

**Methods:**

A comprehensive literature search was undertaken for eligible studies in Embase, PubMed, and Cochrane Library up to Nov 30, 2014. Odds ratios (ORs) and the corresponding 95 % confidence intervals (CIs) were used to measure the strength of the models.

**Results:**

Eight articles (10 case-control studies with 1,502 cases and 1,590 controls) were included in this analysis. Overall, the *AXIN2* rs2240308 polymorphism was associated with a significant increase in the risk of cancer (G allele vs. A allele: OR = 1.21, 95 % CI = 1.05–1.40, *I*^*2*^ = 39.5 % and *P*_*Q*_ = 0.094 for heterogeneity; GG vs. AA: OR = 1.30, 95 % CI = 1.04–1.63, *I*^*2*^ = 35.9 % and *P*_*Q*_ = 0.121 for heterogeneity; GG vs. GA + AA: OR = 1.36, 95 % CI = 1.17–1.58, *I*^*2*^ = 19.5 % and *P*_*Q*_ = 0.263 for heterogeneity). Asian populations showed similar results. Stratified analysis by cancer types indicated that the *AXIN2* rs2240308 polymorphism increases the risk of lung cancer (G allele vs. A allele: OR = 1.36, 95 % CI = 1.17–1.59; GA vs. AA: OR = 1.43, 95 % CI = 1.01–2.02; GG vs. AA: OR = 1.93, 95 % CI = 1.36–2.75; GG + GA vs. AA: OR = 1.65, 95 % CI = 1.18–2.30; GG vs. GA + AA: OR = 1.45, 95 % CI = 1.18–1.79. All *I*^*2*^ < 50 % and *P*_*Q*_ > 0.100 for heterogeneity).

**Conclusions:**

This study showed that the *AXIN2* rs2240308 polymorphism contribute to increasing the risk of cancer, especially lung cancer in Asian populations.

**Electronic supplementary material:**

The online version of this article (doi:10.1186/s12935-015-0219-8) contains supplementary material, which is available to authorized users.

## Introduction

The Wnt signaling pathway plays a crucial role in the development of cancers in humans [1]. The multiprotein destruction complex on the Wnt signaling pathway is organized by glycogen synthase kinase (GSK-3β), adenomatous polyposis coli (APC), and axis inhibition (AXIN). AXIN, a master scaffold protein in the destruction complex, serves as a scaffold protein that facilitates the phosphorylation of β-catenin by GSK-3β and acts as a vital mediator in cellular signaling. *AXIN* is widely considered a negative regulator gene of Wnt/β-catenin signaling and plays an architectural role in integrating incoming signals to downstream effectors, which in turn manifest biological functions [1].

Previous studies indicated AXIN protein expression was correlated inversely with tumor size in breast cancer [2] and increased in colorectal carcinoma (CRC) tissues [3]. The AXIN homologue conduction, also known as AXIL or AXIN2, serves as a scaffolding component of the multiprotein complex and negatively regulates the Wnt/β-catenin pathway [4]. The AXIN2 protein acts as a tumor suppressor in numerous cancers [5, 6]. The *AXIN2* gene has been mapped at human chromosome 17q23-q24, which shows frequent loss of heterozygosity (LOH) in cancers, and mutations in the *AXIN2* gene are associated with colorectal cancer with defective mismatch repair [7, 8]. Some studies focused on the associations between risk of cancer and single nucleotide polymorphisms (SNPs) of the *AXIN2* gene, such as rs3923086, rs3923087, and rs2240308 [9, 10]. The *AXIN2* SNP, Pro50Ser (rs2240308, c.148G > A), results in an amino acid change from a proline to a serine, which is located at exon 1 148 of the *AXIN2* gene, has been widely observed in lung cancer, ovarian cancer and prostate cancer [11–13]. The *AXIN2* rs2240308 polymorphism seems to influence AXIN expression. The function of this SNP is closely associated with Wnt/β-catenin signaling and thus affects carcinogenesis [14].

However, previous literature about the associations between the *AXIN2* rs2240308 polymorphism and risk of cancer has provided inconsistent results. Significant associations have been found in prostate cancer [15] and lung cancer [11, 14, 16], but similar results were not found in ovarian cancer [12], astrocytoma [10], colorectal cancer, and head and neck cancer [16]. Significant racial differences have also been observed in the association between the *AXIN2* rs2240308 polymorphism and the risk of prostate cancer [13, 15]. The objective of this meta-analysis is to evaluate broadly the available evidence on the *AXIN2* rs2240308 polymorphism and risk of cancer, for deriving a more reliable assessment.

## Materials and methods

This meta-analysis was conducted according to the Preferred Reporting Items for Systematic Reviews and Meta-analyses (PRISMA) statement, including the search strategy, selection criteria, data extraction, and data analysis (Additional file 1) [17]. The Venice criteria were used to assess the credibility of the genetic associations [18].

### Identification of eligible studies

We used the following specific combinations of search terms: “axis inhibition protein 2” or “AXIN2” in combination with “polymorphism”, “mutation” or “variant” in combination with “cancer” or “carcinoma” in Embase, PubMed, and Cochrane Library up to Nov 30, 2014. Two investigators (Yifan Sun and Zhitong Wu) conducted an extensive literature search independently for all publications. Articles in reference lists were also hand-searched. Only English-language articles and human studies were searched.

### Inclusion and exclusion criteria

The following criteria were used to choose studies for inclusion: (1) case-control or cohort design studies; (2) studies offering the ability to extract data for calculating the odds ratio (OR), 95 % confidence intervals (CIs), and Hardy-Weinberg equilibrium (HWE); and (3) the DNA genotyping method and the source of the cases and controls were stated in the study. The exclusion criteria were (1) review articles, letters, case reports, editorials, and conference abstracts and (2) family-based studies.

### Data extraction

Two investigators (Yifan Sun and Zhitong Wu) independently extracted data from the eligible studies. The data extracted included the first author’s name, publication date, country, ethnicity, total sample size, cancer type, genotyping method, genotype frequencies of the cases and controls, source of the case group and control group, and the source of specimens of cases that determined genotypes; HWE was calculated from the study data. If the literature did not provide sufficient data, the investigators attempted to contact the author to get the original data.

For the subgroup analysis, the cancer type, ethnicity, genotype method, and source of control were categorized according to the studies. If the data in a study came from different cancers, the study was treated as separate studies in our meta-analysis. To determine the accuracy of the extracted information, the data extracted by the two investigators should have been the same; they checked their data again if there was a dispute. If the two investigators could not reach an agreement, the dispute was submitted to a third reviewer (Hong Xu) to decide.

### Quality score assessment

The quality of the selected studies was assessed by two investigators (Yifan Sun and Zhitong Wu) independently following the criteria predefined by Thakkinstian *et al.* [19]. The criteria were based on the sources of the cases and controls, the total sample size, the cases’ specimens, and the HWE of the controls (Table 1). According to the quality score assessment, a study that scored <10 was classified as “low quality,” a study that scored ≥10 was classified as “high quality”; the lowest score was 0, and the highest score was 15 [20].

**Table 1:** Scale for quality assessment

Criteria	Score
Representativeness of cases	
Selected from population or cancer registry	3
Selected from hospital	2
Selected from pathology archives but without description	1
Not described	0
Representativeness of controls	
Population-based	3
Blood donors or volunteers	2
Hospital-based(cancer-free patients)	1
Not described	0
Specimens of cases determining genotypes	
White blood cells or normal tissues	3
Tumor tissues or exfoliated cells of tissue	0
Hardy-Weinberg equilibrium in controls	
Hardy-Weinberg equilibrium	3
Hardy-Weinberg disequilibrium	0
Total sample size	
≥1000	3
≥500 but <1000	2
≥200 but <500	1
<200	0

### Statistical analysis

We accessed the association between the *AXIN2* rs2240308 polymorphism and risk of cancer by using different comparison models, including an allelic model (G vs. A), a co-dominant model (GA vs. AA, GG vs. AA), a dominant model (GA + GG vs. AA), and a recessive model (GG vs. GA + AA). We defined the GA and GG genotypes as “G carriers.” Unadjusted odds ratios (ORs) and the corresponding 95 % confidence intervals (CIs) were calculated according to the frequencies of genotypes but not by logistic regression because it is difficult to get the all original data from author.

Following the literature [21, 22], heterogeneity was assessed with a chi-square Q test and I-square statistics. If *P*_*Q*_ < 0.1 or *I*^*2*^ > 50 %, we considered the heterogeneity significant, and a random-effects model was conducted using the DerSimonian and Laird method. Otherwise, the summary OR and the corresponding 95 % CI were calculated with the fixed-effects model (the Mantel-Haenszel method). We also carried out a subgroup analysis of ethnicity, genotyping method, source of the controls, hepatitis virus type, liver disease type, and quality assessment score. Galbraith plots analysis was performed for further exploration of the heterogeneity.

HWE in the controls was tested with the chi-square test for goodness of fit, and a *P-*value < 0.05 was considered out of HWE. Sensitivity analysis was conducted to examine such influence by removing studies one by one and recalculating the pooled OR and 95 % CI.

Begg’s funnel plot and Egger’s test were used to investigate the publication bias in the meta-analysis; *P* < 0.05 indicated that the result was statistically significant.

All the tests in this meta-analysis were conducted with STATA software (version 12.0; Stata Corporation, College Station, Texas, USA).

## Results

### Literature selection and study characteristics

**Fig. 1:**
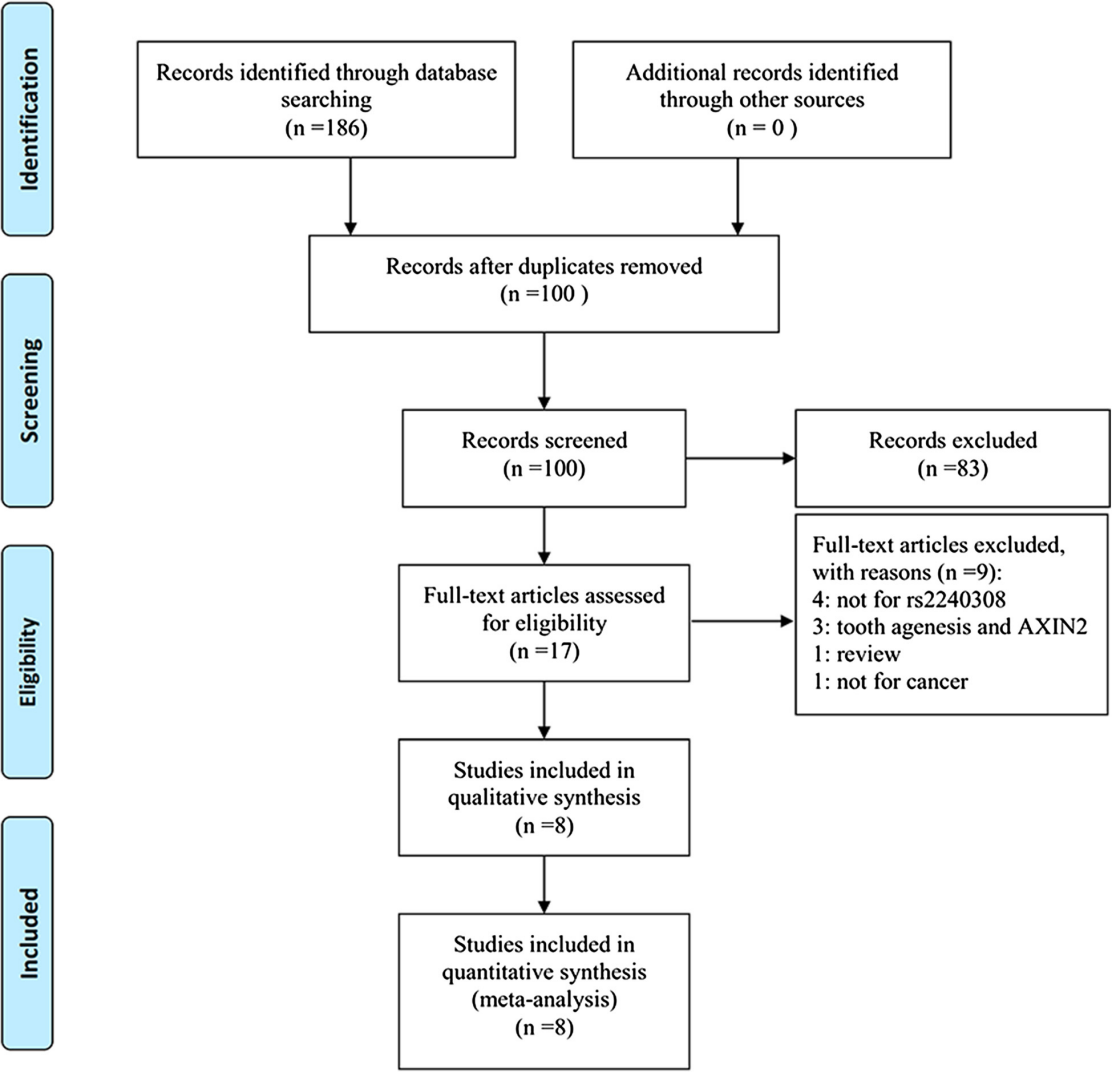
Flow diagram of included studies for this meta-analysis

Figure 1 shows the flow of studies. Based on the search terms, eight articles that included ten case-control studies with 1,502 cases and 1,590 controls were identified as suitable for a meta-analysis [10–16, 23]. Kanzaki H *et al.*’s article [16] was separated into three studies because the researchers studied three cancers. Four articles were on Caucasians, four on Asians; three studies were on lung cancer and two on prostate cancer. The other cancers included colorectal cancer, head and neck cancer, astrocytoma, ovarian cancer, and papillary thyroid carcinoma. HEW was calculated with the genotypes of control population, and one article did not fall into HWE. The quality scores showed the eight studies were “high quality.” Polymerase chain reaction-restriction fragment length polymorphism (PCR-RFLP) was used in 6 studies. The source of the control population was divided into hospital-based (HB) and population-based (PB). The characteristics of the eligible and included studies are listed in Table 2.

**Table 2:** Characteristics of studies included in the meta-analysis

First author	Year	Country	Ethnicity	Case/control	Control select	Cancer	Genotyping method	HWE	Quality scores
Kanzaki H	2006	Japan	Asian	160/110	PB	LC	PCR-RFLP	0.863	12
Kanzaki H	2006	Japan	Asian	113/110	PB	CC	PCR-RFLP	0.863	12
Kanzaki H	2006	Japan	Asian	63/110	PB	H & NC	PCR-RFLP	0.863	11
Gunes EG	2009	Turkey	Caucasian	100/100	HB	LC	PCR-RFLP	0.500	10
Gunes EG	2010	Turkey	Caucasian	100/100	HB	AS	PCR-RFLP	0.500	10
E Pinarbasi	2010	Turkey	Caucasian	84/100	HB	PC	PCR-RFLP	0.882	9
Mostowska A	2014	Polish	Caucasian	282/282	HB	OC	HybProbe	0.546	11
Liu X	2014	China	Asian	56/50	HB	PTC	MALDI-TOF-MS	0.019	6
Liu D	2014	China	Asian	520/555	PB	LC	TaqMan	0.457	14
Ma C	2014	China	Asian	103/100	HB	PC	SNaPshot	0.153	10

*PB* Population–based, *HB* Hospital–based, *LC* lung cancer, *CC* colorectal cancer, *H&NC* head and neck cancer, *As* astrocytoma, *PC* prostate cancer, *OC* Ovarian cancer, *PTC* papillary thyroid carcinoma, *PCR-RFLP* Polymerase chain reaction-restriction fragment length polymorphism, *HWE* Hardy–Weinberg equilibrium in control population

### Allele frequencies in different ethnicities

Allele frequencies in different ethnicities were calculated according to the original data of the studies. The *AXIN2* rs2240308 allele G had higher representation in the controls in Asian populations than in Caucasian populations (63.4 % vs. 58.4 %, *P* = 0.001). On average, the frequencies of GG, GA and AA as a proportion of 1 were 0.39, 0.49, and 0.12, respectively, in Asian controls and 0.34, 0.49, and 0.17 in Caucasian controls; a statistically significant difference was shown between the two ethnicities (*P* = 0.003).

### Meta-analysis results

**Fig. 2:**
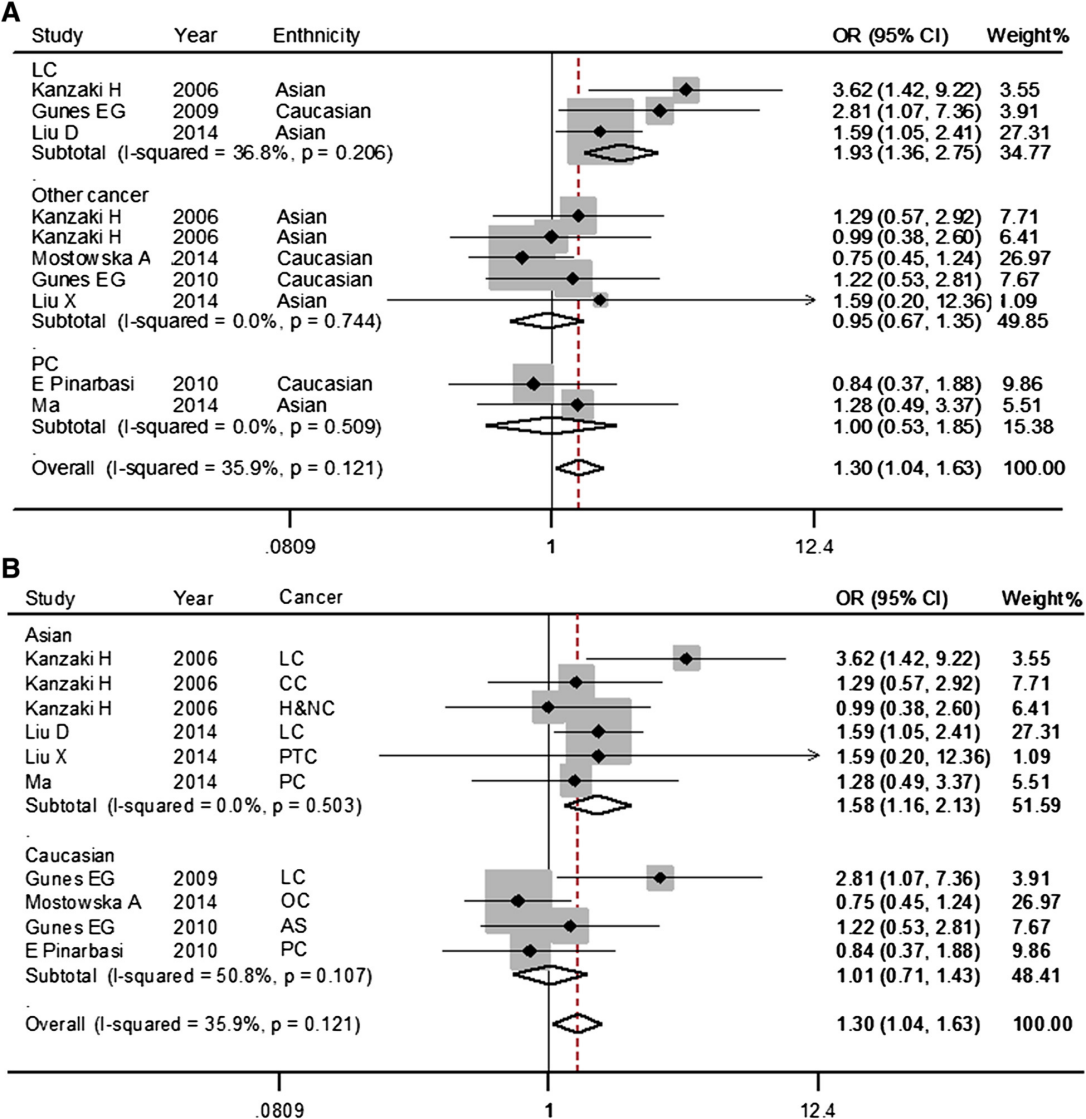
Forest plot for the association between AXIN2 rs2240308 polymorphism and cancer risk stratified by ethnicity (**a**) and cancer type (**b**) in co-dominant model (GG vs. AA) using a fixed-effects mode. The squares and horizontal lines correspond to the study-specific OR and 95 % CI. The diamond represents the summary OR and 95 % CI. LC, lung cancer; PC, prostate cancer; others (colorectal cancer. head and neck cancer, astrocytoma, ovarian cancer, papillary thyroid carcinoma)

The results of the meta-analysis of the *AXIN2* rs2240308 polymorphism and risk of cancer are listed in Table 3. In the random-effects model, the *AXIN2* rs2240308 G allele increased the overall risk of cancer significantly compared with the A allele. Moreover, significant associations were found in a co-dominant model (GG vs. AA) using a fixed-effects model (Fig. 2), as well as a recessive model in the pooled analysis. However, the results for the fixed-effects model indicated a lack of association in the co-dominant model (GA vs. AA) and the dominant model. Based on the previously proposed guidelines [18], the *amount of evidence* was categorized as B since its *n*_*minor*_ is less than 1,000 (*n* = 446); *replication* was assigned to category B with little between-study inconsistency (50 % > *I*^*2*^ > 25 %); and *protection from bias* was graded as category A due to no potential for bias. Therefore, the overall assessment of the association between *AXIN2* polymorphisms and cancer would be *moderate* cumulative evidence (Venice criteria grades = BBA) [18]. Subgroup analysis showed similar results with overall analysis in the Asian populations, PCR-RFLP group, and the studies of quality score assessment ≥ 10.

**Table 3:** Meta-analysis of AXIN2 rs2240308 polymorphisms and cancer risk

Group N		G vs. A			GA vs.AA			GG vs.AA			GG + GA vs.AA			GG vs.GA + AA		
		OR(95 % CI)	*I* ^*2*▲^	*P*_*Q*_^▲^	OR(95 % CI)	*I* ^*2*^	*P*_*Q*_	OR(95 % CI)	*I* ^*2*^	*P*_*Q*_	OR(9 % CI)	*I* ^*2*^	*P*_*Q*_	OR(95 % CI)	*I* ^*2*^	*P*_*Q*_
Overall	10	1.21(1.05–1.40)	39.5	0.094	1.00(0.81–1.14)	12.1	0.332	1.30(1.04–1.63)	35.9	0.121	1.10(0.91–1.42)	27.6	0.362	1.36(1.17–1.58)	19.5	0.263
Ethnicity																
Asian	6	1.32(1.15–1.50)	0.0	0.641	1.11(0.82–1.48)	23.8	0.255	1.54(1.14–2.09)	0.0	0.407	1.32(0.98–1.76)	4.3	0.389	1.48(1.24–1.77)	0.0	0.507
Caucasian	4	1.08(0.83–1.40)	53.7	0.091	0.89(0.65–1.22)	0.0	0.439	1.01(0.71–1.43)	50.8	0.107	0.93(0.69–1.24)	31.0	0.226	1.14(0.88–1.48)	31.2	0.225
Cancer type																
LC	3	1.36(1.17–1.59)	0.0	0.398	1.43(1.01–2.03)	16.2	0.303	1.93(1.36–2.75)	36.8	0.206	1.65(1.18–2.30)	32.0	0.230	1.45(1.18–1.79)	0.0	0.655
PC	2	1.20(0.89–1.62)	63.2	0.099	0.61(0.33–1.11)	0.0	0.587	1.00(0.53–1.85)	0.0	0.509	0.78(0.44–1.38)	0.0	0.872	1.58(0.76–3.28)	67.8	0.078
others	5	0.97(0.83–1.15)	7.3	0.362	0.80(0.60–1.07)	0.0	0.868	0.95(0.67–1.35)	0.0	0.762	0.83(0.63–1.10)	0.0	0.968	1.16(0.91–1.49)	14.5	0.322
Genotyping method																
PCR–RFLP	6	1.25(1.06–1.48)	14.2	0.323	1.09(0.77–1.54)	22.8	0.263	1.48(1.04–2.10)	38.3	0.151	1.23(0.89–1.71)	37.8	0.154	1.40(1.11–1.76)	0.0	0.784
others	4	1.08(0.80–1.46)	65.2	0.013	0.89(0.68–1.16)	12.5	0.330	1.17(0.87–1.57)	41.9	0.160	0.97(0.75–1.24)	20.9	0.285	1.31(0.86–2.00)	65.8	0.034
Source of control																
HB	6	1.11(0.95–1.29)	52.1	0.064	0.80(0.60–1.05)	0.0	0.397	1.02(0.74–1.42)	22.9	0.258	0.86(0.66–1.12)	0.0	0.309	1.29(1.02–1.63)	49.5	0.078
PB	4	1.28(1.11–1.48)	0.0	0.461	1.22(0.88–1.68)	14.9	0.317	1.61(1.17–2.23)	27.5	0.247	1.39(1.03–1.89)	27.2	0.249	1.38(1.14–1.68)	0.0	0.749
Score ≥ 10	8	1.23(1.05–1.45)	46.8	0.069	1.03(0.82–1.30)	24.6	0.233	1.35(1.06–1.71)	34.6	0.178	1.15(0.93–1.42)	37.7	0.129	1.36(1.17–1.59)	29.1	0.191

*N* number of studies, *OR* odds ratio, *CI* confidence interval, *PQ* chi–squared Q test value, *LC* lung cancer, *PC* prostate cancer; others(colorectal cancer. head and neck cancer, astrocytoma, Ovarian cancer, papillary thyroid carcinoma), *PCR–RFLP* Polymerase chain reaction-restriction fragment length polymorphism, *HB* Hospital based, *PB* Population-based, *Score* quality scores

^▲^ *I* ^*2*^(%); If *P*_*Q*_ < 0.1 or *I* ^*2*^ ≥ 50 %, using a random-effects model; If *P*_*Q*_ ≥ 0.1 or *I* ^*2*^ < 50 %,using a fixed- effects model

As shown in Fig. 2 and Table 3, for the stratified analysis by cancer type, significantly increased risk was found among lung cancer in all comparison models; no statistical heterogeneity was discovered. However, a similar significant association was not observed in prostate cancer and other cancers. For the association between *AXIN2* polymorphisms and lung cancer, the *amount of evidence* was categorized as B since its *n*_*minor*_ was above 100 but less than 1,000 (*n* = 114), *replication* was assigned to category B with little between-study inconsistency (*I*^*2*^ < 50 %); and *protection from bias* was graded as category C due to the considerable potential for bias (only 3 studies). Therefore, cumulative evidence for the association between *AXIN2* polymorphisms and lung cancer was categorized as *weak* since there was a C.

### Heterogeneity analysis

For the *AXIN2* rs2240308 polymorphism in the overall population, statistical heterogeneity was discovered only in the allele model, which had *I*^*2*^ values of heterogeneity greater than 50 % and *P*_*Q*_ values lower than 0.100. Then we carried out subgroup analysis according to ethnicity, cancer type, genotype method, source of controls, and quality score assessment. Heterogeneity still existed in some subgroup analysis (*P*_*Q*_ < 0.1, Table 3). We performed a Galbraith plot analysis to confirm the outliers that might cause the heterogeneity, and the results showed that Mostowska A *et al.*[12] study was the outlier in the allele model. We found significantly higher A allele frequencies than other studies. The summary OR value did not change significantly; however, the *I*^*2*^ values were lower than 50 %, and the *P*_*Q*_ value was larger than 0.10 after the study was excluded (OR = 1.287, 95 % CI = 1.146–1.444, *P* = 0.000, *I*^*2*^% = 0.0, *P*_*Q*_ = 0.550 for heterogeneity).

### Sensitivity analysis

The control groups in Liu X *et al.*’s [23] study were out of HWE (Table 2). Thus, this study was excluded to perform a sensitivity analysis for the *AXIN2* rs2240308 polymorphism to the pooled ORs; the significance of all ORs did not change. Further sensitivity analysis was performed by excluding the studies one by one; finally, the corresponding pooled ORs were not qualitatively altered.

### Publication bias

**Fig. 3:**
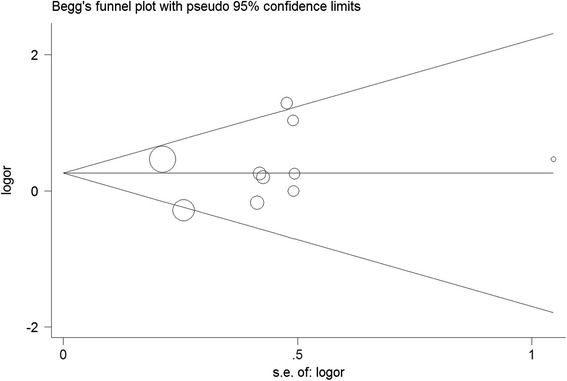
Begg’s funnel plot for contrast in overall analysis in addition model (GG vs. AA). Each point represents a separate study for the indicated association. Size graph symbol by weights. *Log [OR]* natural logarithm of OR. Horizontal line mean effect size

To investigate publication bias in the meta-analysis, corresponding methods, including Begg’s funnel plot and Egger’s test, were conducted. The results showed that no significant publication bias was detected using Begg’s funnel plot in the overall population in all comparison models. The statistical results for Egger’s test also showed evidence of funnel plot symmetry (*P* > 0.05). Figure 3 shows Begg’s funnel plot in the co-dominant model (GG vs. AA, *P*_*Egger’s* test_ = 0.551, 95 % CI = –1.84–3.21).

## Discussion

This is the first meta-analysis of the association between the *AXIN2* rs2240308 polymorphism and risk of cancer. Our results, based on 8 articles that included 10 case-control studies with 1,502 cases and 1,590 controls, suggested that the *AXIN2* rs2240308 polymorphism increases the risk of cancer in a co-dominant model (GG vs. AA) and the recessive model, especially in lung cancer and Asian populations.

Our conclusion is biologically plausible. First, as a tumor suppressor gene, *AXIN2* polymorphisms on the susceptibility of cancer have been found in lung cancer [11], prostate cancer [13], and other cancers [24], while these cancers have all been linked to Wnt signaling [1]. AXIN2 is a transcriptional target of the Wnt signaling pathway. AXIN2 can be unregulated by β-catenin, providing a negative feedback loop to restrain Wnt signaling [25]. Second, a region of *AXIN2* is involved in the APC-binding site, and AXIN and APC are critical for β-catenin regulation [26]. Truncated mutations in APC that eliminate the AXIN-binding site result in human cancer, suggesting that the binding avidity of the AXIN protein for APC affects carcinogenesis. Low expression of AXIN2 was found to be associated with β-catenin accumulation in lung cancer, and epigenetic silencing of AXIN2 leads to β-catenin nuclear accumulation in tumorigenesis [27]. It has also been reported that AXIN2 mutations lead to increased β-catenin concentrations in cancer with defective mismatch repair systems [8]. It is believed that the mutation of *AXIN2* rs2240308 may influence the expression of the AXIN protein, and then dysfunction in these signaling pathways, which play critical roles in carcinogenesis.

In the subgroup analysis by ethnicity, statistical significant associations were found in Asians but not in Caucasians. In fact, it is a widespread phenomenon that the same gene polymorphism plays different roles in risk of cancer among different ethnic subpopulations. Cancer is a complex multigenetic disease, and different genetic backgrounds may contribute to the discrepancy. Moreover, we observed statistically significant differences in allele and genotype frequencies of *AXIN2* rs2240308 between Asians and Caucasians.

To investigate the association between the *AXIN2* rs2240308 polymorphism and risk of cancer in different cancers, we also performed a subgroup analysis by cancer type. The results indicated that the *AXIN2* rs2240308 polymorphism was significantly associated with an increased risk of lung cancer but not in other cancer types. Several possibilities may explain this result. First, in addition to acting as a tumor suppressor protein by negatively regulating Wnt signaling, AXIN also plays a positive role in TGF-β signaling and regulates TGF-β signaling by acting as an adaptor for Smad3, one of the TGF-β effectors [28]. The TGF-β signaling pathway is considered a tumor suppressor and a cancer promoter [29]. Decreased expression of AXIN has been detected in lung cancer tissues [30], while the expression of AXIN2 increases in CRC [3]. These factors suggest that AXIN plays a dual role in the development of different cancers. Second, gene-gene and gene-environmental interactions may lead to the formation of different cancers [31–33]. Third, limited studies and small sample sizes may lead to insufficient statistical power; therefore, we should carefully interpret the negative results.

Since hospital-based controls cannot truly represent the general population, we performed subgroup analysis of the source of the controls. The results revealed that statistical significant associations were observed only in population-based studies, not in hospital-based studies, which suggested that selection bias and recall bias of the study population should be considered when explaining these inconsistent results. A previous study also indicated that hospital-based case-control studies have a high risk of producing unreliable results, and suggested that a methodologically preferable design with an appropriate representative population-based study is crucial to avoid selection bias [34].

Heterogeneity is the most common problem when explaining the results of a meta-analysis. In this meta-analysis, we assessed heterogeneity by using different statistical methods. We found Mostowska A *et al.*’s [12] study was the source of heterogeneity in the allelic comparison model, due to significant higher A allele frequencies compared with other studies. However, the summary OR value did not change significantly after the study was excluded; in addition, all of the other comparison models had no significant heterogeneity (*P*_*Q*_ ≥ 0.1). Therefore, we think that our results were statistically robust, and the sensitivity analysis confirmed this point.

Our meta-analysis had several limitations that must be considered when interpreting the results. Insufficient studies and small sample sizes were the biggest problem. The lack of association in other cancers may also be most likely because of insufficient studies. According to the Venice interim guidelines, the cumulative evidence for the association between *AXIN2* polymorphisms and lung cancer is categorized as *weak*. Therefore, further studies in different ethnicities and cancers should be conducted to strengthen our results. Furthermore, because original data were lacking in the reviewed studies, a more precise analysis was not conducted if individual information including other covariates such as age, sex, and cancer stage became available. Finally, we must pay attention to publication bias when explaining the results even though it was not observed in statistical tests.

## Conclusions

Despite these limitations, our meta-analysis suggested that the *AXIN2* rs2240308 polymorphism increases the risk of cancer, especially in lung cancer and Asian populations. We expect relevant studies to be published in the future to strengthen our conclusion, due to the important role of AXIN2 in Wnt signaling.

## Additional file

Additional file 1:
**PRISMA 2009 Checklist.**
